# The role of impulsivity and perceived availability on cue-elicited craving for alcohol in social drinkers

**DOI:** 10.1007/s00213-012-2747-4

**Published:** 2012-05-26

**Authors:** Harilaos Papachristou, Chantal Nederkoorn, Jan Corstjens, Anita Jansen

**Affiliations:** 1 Faculty of Psychology and Neuroscience, Maastricht University, P.O. Box 616, 6200 MD Maastricht, the Netherlands; 2 U-Center, Epen, the Netherlands

**Keywords:** Cue reactivity, Craving, Alcohol cue exposure, Impulsivity, Response inhibition, Perceived availability

## Abstract

**Rationale:**

Previous research has demonstrated a role for impulsivity and perceived availability of the substance in cue-elicited craving. However, their effects on cue-elicited craving for alcohol are still ambiguous. Most important is that there has been no empirical evidence for the potential interaction of these factors on alcohol craving.

**Objectives:**

The aim of the present study was to examine the effects of response inhibition and perceived availability on cue-elicited craving for alcohol in social drinkers.

**Methods:**

Participants were light to moderate social drinkers (*N* = 75) who were exposed to neutral- and alcohol-related stimuli during a single laboratory session. Response inhibition was assessed with the Stop Signal Task. Participants were randomly assigned to one of two perceived availability groups (*n* = 37, expecting alcohol; *n* = 38, not expecting alcohol).

**Results:**

Overall craving for alcohol was higher in participants who expected alcohol than in those who did not. This finding was statistically significant only in the alcohol condition. Most important is that there was a significant interaction between response inhibition, perceived availability and time on cue-elicited craving. Regardless of the cue type, impulsive people who expected alcohol experience a significant increase in cue-elicited craving relative to impulsive people who did not expect alcohol. This effect was not observed in the non-impulsive groups.

**Conclusions:**

The results clearly show that perceived availability alone and in combination with response inhibition can modulate alcohol cue reactivity. Theoretical explanations and clinical implications of these findings are discussed.

## Introduction

Cue reactivity is a robust phenomenon in the alcohol literature. Alcohol-dependent and heavy social drinkers often report an increase in subjective craving and demonstrate significant physiological reactions to alcohol-related cues (Drummond 2000; Fox et al. 2007; Field and Duka 2002). However, there is still controversy about the origins and nature as well as the factors involved in this phenomenon. For example, cue reactivity has been conceptualized as being either a drug-like appetitive, or a withdrawal-like, or finally a homeostatic response that opposes the unconditioned drug effect (Stewart et al. 1984; Wikler 1948; Siegel 2001). Although there is supportive evidence for all the aforementioned models, most evidence suggests that stimuli associated with alcohol consumption become conditioned incentives, thus eliciting appetitive responses and motivating drinking (Field and Duka 2002; Carter and Tiffany 1999; Drummond 2000).

However, not everybody who drinks alcohol experiences the same levels of cue-elicited craving. Even among alcohol-dependent people, there is evidence that approximately a third does not report craving when exposed to alcohol-related cues (Litt et al. 2000). Apparently, the relationship between cue-elicited craving and alcohol misuse/abuse is complex, and it appears that personality and cognitive factors are involved in it (Papachristou et al. 2012; Wertz and Sayette 2001).

Regarding personality factors, *impulsivity* seems to be a possible candidate. Impulsivity is typically associated with a lack of planning, a difficulty in inhibiting inappropriate behaviour and insensitivity to consequences (Dawe et al. 2004; Dawe and Loxton 2004; Reynolds et al. 2006; Dom et al. 2007). However, most impulsivity measures correlate weakly to each other, which implies that impulsivity is not a unidimensional concept (Dawe and Loxton 2004). A line of research suggests that *response inhibition*, the ability to inhibit a prepotent response, is a distinct psychological process under the general concept of impulsivity (Dawe et al. 2004; Nederkoorn et al. 2009; Papachristou et al. 2012).

A line of research has shown that heavy and dependent drinkers exhibit deficiencies in response to inhibition (Christiansen et al. 2012), though the findings are not always consistent across studies (Fernie et al. 2010; Kamarajan et al. 2005). For example, Colder and O’Connor (2002) reported that high levels of alcohol consumption are associated with deficiencies in response inhibition. Additionally, Rubio et al. (2008) conducted a 4-year follow-up study and reported that performance on the Stop Signal Task, a behavioural measure of response inhibition, predicts the development of alcohol use disorders.

In theory, an impaired response inhibition system could lead to a strong cue-elicited craving for alcohol via a difficulty in inhibiting an appetitive response to a stimulus with strong incentive properties (e.g. an alcohol cue; Dawe and Loxton 2004; Papachristou et al. 2012). Although there is a scarcity of empirical studies in the field, there is some evidence to support the above assumption. Papachristou et al. (2012) reported that response inhibition moderates cue-elicited craving for alcohol in heavy, but not light, social drinkers.

Apart from personality factors, cognitive variables appear also to influence cue-elicited craving. One possible candidate here is the *perceived availability* of the substance. In the present study, this term is identical to what Wertz and Sayette define as “perceived drug use opportunity” or “drug availability, intention to use the drug, and the expectation of experiencing the drug’s effects, assuming at least a minimal desire for drug use” (Wertz and Sayette 2001, cited in p. 4). Among several explanations, the authors propose that the perceived availability of the substance may be an integral component of the conditioned stimulus (e.g. alcohol-related cue). Consequently, substance-related cues may elicit less or even no craving when the substance is perceived to be unavailable because the stimulus complex is not complete (Wertz and Sayette 2001). An alternative explanation could be that throughout the course of substance use/abuse, information regarding drug use opportunity is becoming a distinct conditioned stimulus, which elicits cue reactivity even when physical drug cues are not present (Wertz and Sayette 2001). Consistent with a conditioning account, Field and Cox (2008) also suggest that drug-related cues first elicit an expectation for the use of the substance, which in turn leads to craving. In their opinion, cue-elicited craving is mediated by the perceived availability of the substance (Field and Cox 2008).

Supportive (though tentative) evidence for the role of perceived availability in cue-elicited craving comes from the fact that treatment-seeking inpatients usually report lower levels of cue-elicited craving than continuing substance users (Wertz and Sayette 2001). One reason for this weak response to the substance-related cues could be the perceived unavailability of the substance for the inpatients in the treatment centres (Wertz and Sayette 2001). Additionally, stronger supportive evidence comes from laboratory studies with nicotine-dependent smokers (Field and Cox 2008). Most dependent smokers show an increased craving when exposed to smoking-related cues, whilst self-reported craving significantly decreases when smokers perceive no opportunity to smoke in the near future (Droungas et al. 1995; Juliano and Brandon 1998).

Nevertheless, in the field of alcohol, the findings are not as consistent as in the field of nicotine. Davidson et al. (2003) found that perceived availability does not have an effect on craving for alcohol in non-treatment-seeking alcohol-dependent people. Furthermore, MacKillop and Lisman (2005) reported that when heavy drinkers are exposed to alcohol cues, it is the unavailability and not the availability of alcohol that is associated with higher craving. However, the same authors also reported that in heavy drinkers, unavailability information leads to higher craving for alcohol regardless of the type of cue exposure (alcohol vs. water cues; MacKillop and Lisman 2007). Clearly, there is a need for more research on the role of perceived availability on cue-elicited craving for alcohol in social and dependent drinkers.

The aim of the present study was to examine the role of perceived availability and response inhibition on cue-elicited craving for alcohol in social drinkers. First of all, it is hypothesized that craving for alcohol is higher during alcohol than during water exposure. The increase in craving is expected to be stronger when alcohol is perceived to be available than when it is not. Additionally, it is hypothesized that social drinkers with impaired response inhibition experience higher cue-elicited craving for alcohol than social drinkers with good response inhibition. Finally, it is expected that the effects of perceived availability may be stronger in those social drinkers with impaired response inhibition than in social drinkers with good response inhibition. Patterson and Newman (1993) argue that disinhibited people, even under aversive conditions, focus more and respond more forcefully to salient and rewarding stimuli in the environment than normal people. As mentioned above, perceived availability is considered to be either a salient component of the stimulus complex or a distinct salient conditioned stimulus (CS) on its own. Therefore, for those social drinkers with impaired response inhibition, availability information may be an additional cue (or cue component) to attend and to respond. Consequently, their craving response to this cue may be stronger than the craving response of social drinkers with adequate response inhibition. This interaction may also be able to explain some of the inconsistent findings in the alcohol literature.

## Methods

### Participants

Seventy-five participants (25 men and 50 women) with a mean age of 23.29 years (SD = 5.20 years) volunteered to participate in the study. All of them were recruited from Maastricht University via e-mails and advertisements placed in the university premises. Only participants who were able and willing to consume alcohol were invited to take part in the study. Participants who did not wish to consume alcohol for personal, health, cultural, religious or other reasons could not participate in this experiment. Other exclusion criteria were: (a) having a hepatic disease, (b) being pregnant and (c) receiving any medication that could interact with alcohol. None of the participants had been diagnosed with any substance abuse disorder (apart from smoking tobacco). All participants had to specify their typical type/brand of alcoholic beverage before being invited to the laboratory. At the end of the experiment, participants were rewarded with either a 20€ gift certificate or course credits to fulfil academic requirements.

### Measures

#### Response inhibition

The Stop Signal Task (SST) was used to assess response inhibition (Logan et al. 1997). The task begins with a 500-ms fixation cross presented in the centre of a computer screen. Then, a go trial follows. In each go trial, a square pattern appears either on the left or the right compartment of the screen and participants are required to push as quickly as possible the left or right “shift” button, respectively. However, in 25 % of the go trials, an acoustic stop signal (1,000-Hz tone) is heard after the go signal, indicating that the participants must withhold their response. The stop signal delay initially occurs at 250 ms after the go signal presentation, but throughout the task it changes according to the participant’s performance. These adjustments enable the participants to successfully inhibit their responses at approximately 50 % of the stop trials.

First, participants performed three practice blocks of respectively 6, 12 and 24 trials. Next, four test blocks of 64 trials each followed. The inter-trial interval was 1,000 ms. The dependent variable of interest is the stop signal reaction time (SSRT). A higher SSRT means that it takes more time for a participant to inhibit a prepotent response; thus, it is an index of impaired response inhibition.

#### Craving

Craving was assessed with two 100-mm visual analogue scales. Participants were asked to indicate (a) their desire to consume alcohol—“How much do you feel like drinking alcohol right now?”—and (b) their urge to drink alcohol—“How strong is your urge to drink alcohol right now?” Visual analogue scales have been used in many alcohol-related studies and have been found to be valid and reliable indicators of craving for alcohol (Juliano and Brandon 1998; Kozlowski et al. 1996).

#### Alcohol Use Identification Test

The Alcohol Use Identification Test (AUDIT) is a reliable screening tool for identifying those people whose alcohol consumption is excessive and reaches problematic levels (Saunders et al. 1993). It consists of ten multiple-choice items which assess hazardous alcohol consumption, alcohol dependence symptoms and harmful alcohol use (Saunders et al. 1993).

#### Timeline Follow-back Questionnaire

Alcohol use was also assessed with the Timeline Follow-back Questionnaire. When completing this questionnaire, participants are required to provide a detailed description of the frequency and quantity of their alcohol consumption (expressed in standard units or drinks) over the last 30 days (Sobell and Sobell 1990). The sum score of standard drinks was used as an index of each participant’s alcohol consumption.

### Procedure

Ethical approval was obtained from the Ethical Committee of the Psychology Faculty of Maastricht University. Participants took part in one individual testing session arranged between 1230 and 1900 hours.

After signing the informed consent form, participants had to complete a brief demographic questionnaire. Having done this, they were presented with the Timeline Alcohol Questionnaire and the AUDIT and then were instructed to perform the SST. Following this, participants were first offered a small amount of water to control for thirst and then were explicitly told that they had been randomly allocated to either the “expecting alcohol” or the “not expecting alcohol” conditions. Unlike the latter group, the former would be offered a glass of their typical alcoholic beverage at the end of the experiment. Participants were explicitly asked if they had understood the implications of this allocation (e.g. “I would like to know if you have understood the condition in which you are in. Are you going to have a drink of alcohol at the end of the experiment or not?”). When the participants confirmed that they had understood the condition they were in, they were told that they would be exposed first to water and then to alcohol cues. As Rohsenow and Niaura (1999) recommend, the order of the cue exposure conditions was not counterbalanced to avoid carryover effects.

At the beginning of the water exposure, participants were presented with a tray containing a commercially labelled bottle of spring water, an empty glass for water and a small glass containing 1.5 ml of water. After the water exposure, a 5-min break followed, during which the participants were offered some magazines to read (without alcohol-related advertisements) and left alone to relax. Immediately after the break, participants were again offered a small amount of water to control for thirst and were once more asked to report the drinking condition they were in. Then, the alcohol cue exposure started. Like the water exposure, participants were confronted with a commercially labelled bottle of their typical alcoholic beverage, a proper glass for the alcoholic beverage, a small glass containing 1.5 ml of this alcoholic beverage and, if necessary, a bottle opener.

Each cue exposure condition lasted 20 min. Participants had to sit down in front of a table facing the wall. The experimenter first ensured that the lab was quiet and the lights dimmed and then sat down on a chair behind the participant and instructed them what to do with the cues. During each cue exposure condition, craving was assessed five times, once at the beginning (baseline) and then every 5 min. The complexity of the cue exposure increased across time (Greeley et al. 1993). For example, during the first 5 min, participants were exposed only to imagery and visual cues. However, in the next 5 min, they were also exposed to olfactory cues. Following this, taste was also included by having participants immerse their fingers into the beverage (water or alcohol) and touch their mouth and tongue with those fingers. Finally, during the last 5 min, they had also to drink 1.5 ml of either water or alcohol (most intense exposure).

After the alcohol cue exposure, half the participants were offered a glass of their typical alcoholic beverage (“expecting alcohol” condition) whilst the other half were not offered alcohol (“not expecting alcohol” condition). For safety reasons, the blood alcohol level of the former group was monitored with a breath analyzer before they left the laboratory. All participants signed up on a debriefing list in order to receive a debriefing e-mail after the experiment had ended. Finally, they were thanked and rewarded for their participation in the experiment.

### Statistical analysis

Participants were divided into two perceived availability groups (expecting alcohol, *n* = 37; not expecting alcohol, *n* = 38). Perceived availability condition was the between-subjects factor in the analysis. The SSRT and AUDIT were centred and entered as covariates into the analysis. Craving for alcohol was the dependent variable and was calculated by averaging the scores in the two visual analogue scales for each participant. For the purpose of the present analysis, only the last time point of the cue exposure was chosen (most intense cue exposure). A three-way 2 (cue: water vs. alcohol) × 2 (time: baseline vs. exposure × 2 (perceived availability: expecting vs. not expecting alcohol) mixed ANCOVA was performed on craving for alcohol. In addition, interactions with the covariates (SSRT and AUDIT) were tested. When further analysis was required, a median split was conducted on the SSRTs in order to classify social drinkers as being either good or impaired in response inhibition. Greenhouse–Geisser correction was used when Mauchly’s test of sphericity was significant.

## Results

### General characteristics of the sample

Participants had consumed on average 47.8 (SD = 44.57) standard alcoholic drinks in the 30 days before participation in the experiment; their mean AUDIT score was 7.72 (SD = 4.16).

### Differences between perceived availability conditions

There was no significant difference in baseline craving levels, AUDIT scores, amount of standard drinks/month and SSRTs between people who expected and people who did not expect alcohol in the present experiment (Table 1). Table 2 depicts the correlations between SSRT, AUDIT scores, craving scores after alcohol cue exposure and standard drinks/month for each perceived availability condition.

**Table 1:** Differences (mean and standard deviation) between perceived availability conditions

Variables	Expecting alcohol (*n* = 37)	Not expecting alcohol (*n* = 38)
Craving levels at water baseline	2.51 (1.67)a	2.20 (1.87)b
SSRT	203.84 (32.03)a	202.97 (33.22)b
AUDIT	7.76 (4.13)a	7.68 (4.24)b
Standard drinks/month	46.08 (46.04)a	49.47 (43.66)b

Means sharing similar lowercase letters within a row differ at *p* < .05

*SSRT* Stop Signal Reaction Time, *AUDIT* Alcohol Use Identification Test

**Table 2:** Correlations between after SSRT, AUDIT, standard drinks/month and craving after alcohol cue exposure for each perceived availability condition

Perceived availability condition		SSRT	AUDIT	Standard drinks/month	Craving after alcohol cue exposure
Expecting alcohol (*n* = 37)	SSRT		.11	.26	.33^a^
	AUDIT	.11		.78^b^	.23
	Standard drinks/month	.26	.78^b^		.28
	Craving after alcohol cue exposure	.33^a^	.23	.28	
Not expecting alcohol (*n* = 38)	SSRT		−.17	−.15	−.22
	AUDIT	−.17		.72^b^	.49^b^
	Standard drinks/month	−.15	.72^b^		.34^a^
	Craving after alcohol cue exposure	−.22	.49^b^	.34^a^	

*SSRT* Stop Signal Reaction Time, *AUDIT* Alcohol Use Identification Test

^b^Correlation is significant at the .01 level (two-tailed)

^a^Correlation is significant at the .05 level (two-tailed)

### The effect of cue exposure

**Fig. 1:**
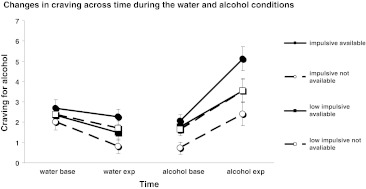
Craving levels across time during the water and alcohol conditions when alcohol was perceived available or not available and when people were impaired (+1 SD above the mean) or good (−1 SD below the mean) in response inhibition

There was a main effect of the type of cue (water, alcohol) on the overall craving for alcohol (baseline + exposure): *F*(1,67) = 21.82, *p* < .001 (Fig. 1). Craving for alcohol is higher in the alcohol than in the water condition (Fig. 1). Moreover, a significant effect of time was found, *F*(1,67) = 28.83, *p* < .001. Overall, craving for alcohol is higher after cue exposure than at baseline (Fig. 1). Finally, there was a significant two-way interaction of the type of cue (water, alcohol) × time (baseline, exposure) on craving: *F*(1,67) = 81.24, *p* < .001. Inspection of Fig. 1 indicates that the pattern of change in craving levels across time differs between the water and the alcohol conditions. After the water exposure, craving for alcohol is significantly reduced compared to baseline levels: *t*(74) = −4.38, *p* < .001. On the other hand, craving for alcohol significantly increases after exposure to alcohol cues relative to baseline levels: *t*(74) = 9.68, *p* < .001. Finally, there was a main effect of AUDIT on craving: *F*(1,67) = 12, 62, *p* = .001. Scoring higher in AUDIT is associated with a higher craving for alcohol in the present study. However, none of the interactions with AUDIT was significant (cue type × time × AUDIT: *F*(1,67) = .01, ns; cue × AUDIT: *F*(1,67) = 1.57, ns; time × AUDIT: *F*(1,67) = 2.09, ns).

### The effect of the perceived availability

A significant main effect of perceived availability (expecting vs. not expecting alcohol) on craving for alcohol was found: *F*(1,67) = 5.33, *p* < .05. Overall, social drinkers who expected to consume alcohol at the end of the experiment experience a higher craving (baseline + exposure) during both the water and the alcohol conditions than those participants who perceived alcohol to be unavailable in the laboratory. The effect of availability was qualified by a significant two-way interaction between the type of cue (water, alcohol) and perceived availability (expecting vs. not expecting alcohol) on craving for alcohol: *F*(1,67) = 5.55, *p* < .05. Independent-samples *t* tests indicated that craving levels during the water condition (baseline + exposure) do not differ significantly between the two perceived availability groups: *t*(73) = 1.35, ns. However, during the alcohol condition (baseline + exposure), social drinkers who expected alcohol experience a significantly higher craving than those participants who did not expect alcohol: *t*(73) = 2.48, *p* < .05 (Fig. 1). Finally, none of the interactions with AUDIT was significant (perceived availability × AUDIT: *F*(1,67) = 0.81, ns; cue type × perceived availability × AUDIT: *F*(1,67) = 0.57, ns).

### Effect of response inhibition

There was no significant main effect of response inhibition on craving: *F*(1,67) = 0.00, ns. Furthermore, there was no significant interaction between response inhibition and perceived availability on craving: *F*(1,67) = 2.8, *p* = .099. Similarly, the interactions response inhibition × AUDIT (*F*(1,67) = 0.00, ns) and response inhibition × perceived availability × AUDIT (*F*(1,67) = 0.16, ns) on craving were not significant. However, there was a significant three-way interaction between time (baseline, exposure) × response inhibition × perceived availability (expecting, not expecting alcohol) on craving for alcohol: *F*(1,67) = 5.51, *p* < .05. Yet, the four-way interaction time × response inhibition × perceived availability × AUDIT on craving was not significant: *F*(1,67) = 0.062, ns. To analyse the three-way interaction further, a median split was conducted on SSRTs and participants were divided into good and impaired in response inhibition. A two-way ANCOVA time × perceived availability with AUDIT as the covariate was performed on craving for each response inhibition group, respectively. The analysis showed that the interaction time × perceived availability on craving was not significant for those participants with good response inhibition: *F*(1,34) = 0.29, ns (Fig. 1). On the other hand, the same interaction was significant for those social drinkers with impaired response inhibition: *F*(1,35) = 6.99, *p* < .05 (Fig. 1). Only in the latter group was the difference in craving between exposure to any cue (water or alcohol) and baseline different between the two perceived availability conditions (expecting vs. not expecting alcohol). Further analysis within the impaired response inhibition group (and whilst controlling for AUDIT) showed that the main effect of time (baseline, exposure) was significant for those social drinkers who were expecting to consume alcohol at the end of the experiment (*F*(1,18) = 11.76, *p* = .003), but non-significant for those social drinkers who could not drink at the end of the experiment (*F*(1,16) = .23, ns; Fig. 1). Inspection of Fig. 1 shows that regardless of the cue type, social drinkers with impaired response inhibition who expected to consume alcohol experience their highest craving during exposure (i.e. higher increase in craving during alcohol exposure and less decrease of craving during water exposure).

The three-way interaction between cue (water, alcohol) × response inhibition × perceived availability (expecting vs. not expecting alcohol) was non-significant: *F*(1,71) = 2.14, ns. Similarly, the four-way interaction cue (water, alcohol) × time (baseline, exposure) × response inhibition × perceived availability (expecting vs. not expecting alcohol) on craving was not significant: *F*(1,71) = 0.18, ns. Finally, the four-way interaction cue type × response inhibition × perceived availability × AUDIT on craving (*F*(1,67) = 0.27, ns) and the five-way interaction cue type × time × response inhibition × perceived availability × AUDIT on craving (*F*(1,67) = 2.99, ns) were not significant.

## Discussion

The aim of the present study was to investigate the effects of response inhibition and perceived availability on cue-elicited craving for alcohol in social drinkers. It was hypothesized that cue-elicited craving for alcohol is higher during the alcohol than during the water cue exposure and that this difference in craving levels is larger in participants who expect to consume alcohol relative to those who do not. Additionally, it was assumed that people with impaired vs. good response inhibition react more to alcohol cues. Finally, it was expected that the effects of perceived availability are more intense in social drinkers with impaired vs. unimpaired response inhibition. The results confirmed most of the above hypotheses.

First of all, it was found that alcohol cues elicit stronger craving than neutral (water) cues. This finding verifies our first hypothesis and is in line with the cue reactivity literature (Drummond 2000; Carter and Tiffany 1999). Most important is that a significant interaction between response inhibition, perceived availability and time on craving for alcohol was reported, independent of cue type (water or alcohol). When the response inhibition levels are sufficient, perceived availability has no effect on craving for alcohol. However, when the response inhibition mechanisms are impaired, participants who perceive alcohol to be available experience a significant increase in cue-elicited craving relative to participants who do not expect alcohol. The craving of the latter impulsive group does not change significantly from baseline levels. This is a totally new finding in the cue reactivity literature and therefore awaits further replication. However, it is of primary theoretical and clinical importance because it demonstrates the impact of the combination of personality and cognitive factors on alcohol cue reactivity.

This finding partially affirms our last hypothesis that people with impaired response inhibition are more reactive to their environment when they consider alcohol to be available than unavailable. Disinhibited people may be more sensitive to salient cues (Patterson and Newman 1993; Dawe et al. 2004; Dawe and Loxton 2004) and, as the present results also illustrate, to the absence of these cues. For these participants, perceived availability may be a salient cue to respond, and therefore, their craving for alcohol would be stronger when this cue is present than when it is absent.

It could also be argued that for impulsive people, perceived availability is not part of the physical alcohol stimulus (CS) because it affects craving during both the water and the alcohol cue exposures. Rather, it seems to act as a distinct CS (Wertz and Sayette 2001; Juliano and Brandon 1998). It is possible, for example, that availability knowledge always precedes a drinking episode. As a result of the association with the rewarding effects of alcohol, availability information may itself become a CS that triggers craving for alcohol regardless of the presence of proximal alcohol cues (Juliano and Brandon 1998). Further evidence supporting this idea comes from the fact that specific reactions to alcohol-related cues do not differ between impulsive and non-impulsive participants in the two perceived availability groups because the interaction between cue type, time, perceived availability and response inhibition on craving is not significant. The lack of interaction excludes alternative explanations, such as perceived availability being a necessary component of the CS complex or an occasion setter (Juliano and Brandon 1998; Wertz and Sayette 2001).

The significant main effect of perceived availability may also indicate that the knowledge that alcohol is available may serve as a distinct CS (Wertz and Sayette 2001; Juliano and Brandon 1998). However, the main effect of availability in the present study is qualified by the significant interaction between cue type and perceived availability, which demonstrates that the difference in overall craving (baseline + exposure) between the two groups reaches statistical significance only during the alcohol condition. Nevertheless, it could still be argued that perceived availability serves as a weak CS that causes the non-significant difference in overall craving between the two perceived availability groups during the water condition. In the alcohol condition, however, the combination of the physical alcohol cues with perceived availability may result in a significant difference in overall craving between the groups. This explanation appears to be consistent with Drummond’s (2000, cited in p. 132) concept of a “cue cascade…in which each cue increases…the salience of the next cue”.

Therefore, it appears that perceived availability of the substance may serve as a more distal and distinct CS that acts in combination with other factors to increase craving. For example, it may have a weak effect on craving when presented with neutral stimuli, but a stronger effect when followed by alcohol-related cues because it amplifies their salience (Drummond 2000). Additionally, it may increase craving independent of cue type, but only in combination with high impulsivity levels.

Nevertheless, alternative explanations cannot be excluded. For example, one methodological limitation of the present study was that the water and the alcohol conditions were not counterbalanced in order to avoid transfer of reactivity to the water cues. The alcohol condition was always presented second in order; hence, it was always closer to the end of the experiment, which in turn signals temporal proximity to alcohol drinking for those social drinkers who expected alcohol. Consequently, it is possible that the main effect of availability becomes more pronounced as the time to delivery of the alcohol draws closer. According to this interpretation, availability is an independent cue that does not interact with the alcohol cue.

The clinical significance of the present results should not be underestimated. Alcohol is readily available in most places in Western countries, except for the addiction clinics. People with alcohol problems do not have access to the substance when they undergo inpatient treatment. Moreover, craving/desire for alcohol is often used as an index of the treatment progress of the patient. However, the present study reveals that personality aspects in combination with cognitive factors can modulate craving for alcohol and present a faulty image of the progress of the patient in the clinic. For example, it could be that the more impulsive inpatients who perceive alcohol to be unavailable in the clinic demonstrate no significant craving whilst being in treatment. However, the same people, when being outside the clinic, could experience serious craving for alcohol even in the absence of physical alcohol cues only because they know that alcohol is available. The present data have also consequences for cue exposure treatment in which patients are exposed to alcohol cues. The goal of this treatment is to have participants crave as much as possible whilst the drinking response is prevented. This procedure should extinguish the craving response to the drinking cues (Conklin and Tiffany 2002). However, if the patient knows that there is no opportunity to drink, their craving might be lower and extinction is less likely to happen. In addition, the present data suggest that it is important for impulsive people to expose themselves also to the availability of alcohol. When not extinguished, this cue, which is very salient after leaving an inpatient clinic, might induce strong feelings of craving and possibly trigger a relapse in impulsive drinkers.

In the present study, we did not find a modulating effect of response inhibition in cue-elicited craving for alcohol, which seems to be in disagreement to our hypothesis and the results of our earlier study (Papachristou et al. 2012). In our previous study, we reported a modulating effect of response inhibition on cue-elicited craving for alcohol in heavy (mean AUDIT score = 12.1), but not in light drinkers (mean AUDIT score = 5.81; Papachristou et al. 2012). In the present study, our sample consists of light to moderate social drinkers (mean AUDIT score = 7.72), and this difference in the drinking status of the samples could be a reason for the discrepancy in the findings between the two studies.

The drinking status is also one of the major methodological differences between the present study and earlier alcohol studies on perceived availability (MacKillop and Lisman 2005, 2007). In both studies by MacKillop and Lisman (2005, 2007), participants were heavy drinkers who had to consume at least 20+/14+ standard drinks per week for men/women, respectively (MacKillop and Lisman 2005, 2007). In the present study, we did not include such a criterion for participation. Perhaps, the effects of availability are different for different types of drinkers, but only empirical research can investigate this hypothesis.

The drinking status (light to moderate drinkers) and the gender differences (the majority of the present sample consisted mostly of female rather than male participants) in our sample limit the generalization of these findings. Therefore, the results should be considered to be preliminary and more research should be conducted on the same topic perhaps with different types of drinkers (heavy, people with alcohol use disorders) and samples with different gender proportions. In any case, the present findings are more in agreement with earlier nicotine studies in which perceived availability of tobacco was found to increase craving (Field and Cox 2008; Carter and Tiffany 2001; Juliano and Brandon 1998; Droungas et al. 1995). Unlike the earlier nicotine studies, however, the present data illustrate that perceived availability serves as a distinct CS that acts in combination with personality aspects such as impulsivity to increase craving. Although this is an entirely new finding and should be subject to replication, its clinical implications should not be ignored. More importantly, the results clearly show that a variety of factors and their combination affect cue reactivity, which may partly explain the lack of and the variability in cue reactivity observed in clinical and non-clinical populations (Litt et al. 2000; Carter and Tiffany 1999).
